# Preliminary verification of the anti-hypoxia mechanism of Gentiana straminea maxim based on UPLC-triple TOF MS/MS and network pharmacology

**DOI:** 10.1186/s12906-022-03773-0

**Published:** 2022-11-25

**Authors:** Xiu mei Kong, Dan Song, Jie Li, Yi Jiang, Xiao ying Zhang, Xiao Jun Wu, Ming juan Ge, Jiao jiao Xu, Xiao min Gao, Qin Zhao

**Affiliations:** 1 Joint Laboratory for Research on Active Components and Pharmacological Mechanism of Tibetan Materia Medica of Tibetan Medical Research Center of Tibet, School of Medicine, Xizang Minzu University, Xianyang, 712082 Shaanxi China; 2 Xianyang Hospital of Yan’an University, Xianyang, 712000 Shaanxi China; 3 Engineering Research Center of Tibetan Medicine Detection Technology, Ministry of Education, Xizang Minzu University, Xianyang, 712082 Shaanxi China

**Keywords:** G.s Maxim’s ethyl acetate extraction, Anti- hypoxia, Network pharmacology, Inflammatory, HIF-1/NF-κB pathway

## Abstract

**Background:**

Anoxia is characterized by changes in the morphology, metabolism, and function of tissues and organs due to insufficient oxygen supply or oxygen dysfunction. *Gentiana straminea* Maxim (G.s Maxim) is a traditional Tibetan medicine. Our previous work found that G.s Maxim mediates resistance to hypoxia, and we found that the ethyl acetate extract had the best effect. Nevertheless, the primary anti-hypoxia components and mechanisms of action remain unclear.

**Methods:**

Compounds from the ethyl acetate extraction of G.s Maxim were identified using UPLC-Triple TOF MS/MS. Then Traditional Chinese Medicine Systematic Pharmacology Database was used to filtrate them. Network pharmacology was used to forecast the mechanisms of these compounds. Male specific pathogen-free Sprague Dawley rats were randomly divided into six groups: (1) Control; (2) Model; (3) 228 mg/kg body weight Rhodiola capsules; (4) 6.66 g/kg body weight the G.s Maxim’s ethyl acetate extraction; (5) 3.33 g/kg body weight the G.s Maxim’s ethyl acetate extraction; (6) 1.67 g/kg body weight the G.s Maxim’s ethyl acetate extraction. After administering intragastric ally for 15 consecutive days, an anoxia model was established using a hypobaric oxygen chamber (7000 m, 24 h). Then Histology, enzyme-linked immunosorbent assays, and western blots were performed to determine these compounds’ anti-hypoxic effects and mechanisms. Finally, we performed a molecular docking test to test these compounds using Auto Dock.

**Results:**

Eight drug-like compounds in G.s Maxim were confirmed using UPLC-Triple TOF MS/MS and Lipinski’s rule. The tumor necrosis factor (TNF) signaling pathway, the hypoxia-inducible factor 1 (HIF-1) signaling pathway, and the nuclear factor kappa-B (NF-κB) signaling pathway was signaling pathways that G.s Maxim mediated anti-anoxia effects. The critical targets were TNF, Jun proto-oncogene (JUN), tumor protein p53 (TP53), and threonine kinase 1 (AKT1). Animal experiments showed that the ethyl acetate extraction of G.s Maxim ameliorated the hypoxia-induced damage of hippocampal nerve cells in the CA1 region and reversed elevated serum expression of TNF-α, IL-6, and NF-κ B in hypoxic rats. The compound also reduced the expression of HIF-1α and p65 and increased the Bcl-2/Bax ratio in brain tissue. These findings suggest that G.s Maxim significantly protects against brain tissue damage in hypoxic rats by suppressing hypoxia-induced apoptosis and inflammation. Ccorosolic acid, oleanolic acid, and ursolic acid had a strong affinity with core targets.

**Conclusions:**

The ethyl acetate extraction of G.s Maxim mediates anti-hypoxic effects, possibly related to inhibiting apoptosis and inflammatory responses through the HIF-1/NF-κB pathway. The primary active components might be corosolic, oleanolic, and ursolic acids.

**Supplementary Information:**

The online version contains supplementary material available at 10.1186/s12906-022-03773-0.

## Introduction

In anoxia, there are abnormal changes in tissues and organs’ morphology, metabolism, and function due to insufficient oxygen supply. This phenomenon leads to stress responses, including tachypnea, tachycardia, and hypertension. When severe hypoxia occurs, histocyte edema, autolysis, and other phenomena may occur, which may cause irreversible damage to the heart and brain, resulting in dysfunction or even failure. Hypoxia occurs at high altitudes and in several pathological situations, including severe asthma, anesthesia, stroke, and cardiovascular injury [1, 2]. Oxygen consumption exceeds physiological mobilization capacity during strenuous exercise and excessive labor, which may also lead to insufficient relative oxygen supply. A growing body of evidence suggests that hypoxia adversely affects vital organs such as the brain [3]. For these reasons, it is essential to identify anti-hypoxia injury medications.

*Gentiana straminea* Maxim (G.s Maxim, translated as “Jie ji ga bao” in Tibetan) is a perennial herbaceous plant of Gentianaceae. It is a traditional Tibetan medicine with more than 2000 years of history. The root of G.s Maxim macrophylla has anti-inflammatory activity and analgesic effects [4–6]. It is often used to treat gastroenteritis, hepatitis, and cholecystitis [7, 8]. Our previous work found that G.s Maxim mediates resistance to hypoxia, and we demonstrated the protective effect of ethanol extracts from G.s Maxim against lung and heart damage in rats at high altitudes [9–11]. We found that the ethyl acetate extract of G.s Maxim had the best effect [12]. It is safer to use G.s Maxim for anti-hypoxia, with few side effects [1, 13]. However, the primary anti-hypoxia components and mechanisms of action remain unclear.

LC-MS/MS is one of the best methods to identify compounds, and network pharmacology is a new discipline in line with the characteristics of traditional Chinese medicine research [14–16]. Therefore, this study identified compounds in the ethyl acetate extracts of G.s Maxim using UPLC-Triple TOF MS/MS. The network pharmacology method predicted the active components, core targets, and action pathways. We established a high-altitude hypoxia rat model and used molecular docking technology to verify the prediction results**.** These study results will offer an opportunity to deepen the understanding of the anti-hypoxia pharmacological mechanisms associated with the ethyl acetate extracts of G.s Maxim.

## Methods

### Drugs and reagents

The dried root that was naturally air-dried from G.s Maxim was purchased from Qamdo Tibetan Hospital (Tibet, China). Rhodiola capsules were obtained from Rhodiola Research and Development Center, Xizang Military Region. TNF-α, IL-6, and NF-κB enzyme-linked immunosorbent assay kits were obtained from Boster Biological Technology (Pleasanton, USA). Primary antibodies for Bcl-2, Bax, HIF-1, p65, and β-tubulin were purchased from Immunoway (Plano, USA).

### Ethyl acetate extraction

A total of 100 g G.s Maxim was added to 500 mL of 95% ethanol for 24 h using a heating reflux device, boiled the material for one hour, and repeated three times. We used a vacuum rotary evaporator (Heidolph, Germany) to evaporate the ethanol and collect the extract. The extraction rate was 10%. Finally, we added double-distilled water to dissolve the resulting drug extract completely.

Extraction was according to the order of polarity of organic solvents; the ethanol extraction of G.s Maxim was extracted with petroleum ether, ethyl acetate, and water-saturated N-butanol solution. After rotary evaporation under reduced pressure at 70 °C, we vacuum freeze-dried the extracts (LGJ-10, China) to obtain powder from each extraction for further use.

### Compound identification using UPLC-triple TOF MS/MS

#### Samples preparation

To 0.2 g of the ethyl acetate extract, we added 5 mL 50% methanol-aqueous solution, let it sit for 4 h, then subjected it to ultrasound at 40 °C for 40 min. The supernatants were placed in 1-mL centrifuge tubes, centrifuged at 1300 r/min for 10 min, and passed through 0.22-μm ultrafiltration membranes (Millipore, Bedford, MA, USA). Finally, the material was placed in 1.5-mL automatic sampling tubes. The blank control samples were obtained under the same conditions.

#### UPLC-triple TOF MS/MS condition

Reverse-phase analysis was performed on a UPLC Nexera system (Shimadzu, Japan) using an ACQUITY UPLC CSH C18 column (2.1 mm × 100 mm, 1.7 μm) (Waters, USA) containing a binary pump, a column oven, and an ESI ionization source. The flow rate was 0.3 mL/min, with mobile phase A composed of 0.1% formic acid and mobile phase B composed of acetonitrile. A gradient elution achieved sample separation: 0.01–15 min, 95–75% A; 15–37.1 min, 75–95% A; 37.1–40 min, 95% A. The mobile phase’s aqueous part pH (0.1% formic acid in H_2_O) was fixed at 2.47.

The column temperature was set to 40 °C, and the injection volume was 2 μL for each analysis. The samples were filtered through a 0.22-μm ultrafiltration membrane before injection.

Mass spectrometric analysis was performed on a Triple TOF® 5600 System (AB SCIEX, USA) in positive and negative ion modes. The source conditions were as follows: spray voltage of 5500 V in ESI (+) and − 4500 V in ESI (−), nebulizing gas at 50 psi, heating gas at 50 psi, curtain gas™ at 40 psi, and heater temperature at 500 °C. The declustering potential was 100 V. MS, and the scan range was 50–1000 (m/z). The mass spectra results were analyzed using Peakview data processing software.

### Network pharmacology analysis

#### Identification of drug-like compounds (DLCs)

The compounds identified using UPLC-Triple TOF MS/MS were screened using the traditional Chinese medicine systems pharmacology (TCMSP) database (http://tcmspw.com/). Bioactive components with oral bioavailability (OB) ≥ 15% and drug-likeness (DL) index ≥0.18 were selected for subsequent analysis.

#### Targets related to DLCs and anoxia

The target proteins of bioactive components in the ethyl acetate extraction were retrieved from the TCMSP database. Search for disease-related targets in the Gene Cards database (https://www.genecards.org/) [17] by keyword “anoxia.” The target proteins were standardized in UniProt (http://www.uniprot.org/). We recorded the duplication of drug and disease targets, then designated them as the anti-hypoxia target of the G.s Maxim.

#### Protein-protein interaction (PPI) analysis

Targets identified in section 2.4.2 were uploaded into the STRING database (https://string-db.org/) [18] to perform PPI analysis, focusing on co-expression and co-localization. Cytoscape (http://www.cytoscape.org/, version 3.8.2) was used to analyze the PPI network, and the core anti-hypoxia targets of the G.s Maxim’s ethyl acetate extraction.

#### Gene ontology and pathway enrichment analysis

Gene ontology (GO) analysis and Kyoto Encyclopedia of Genes and Genomes (KEGG)-pathway enrichment was built using the DAVID Bioinformatics Resources (https://david.ncifcrf.gov/summary.jsp) [19]. Its targets with the involved pathways were obtained using enrichment analysis and explored the potential biological effects for the G.s Maxim’s ethyl acetate extraction targets.

### Animals and treatments

Male specific pathogen-free Sprague Dawley rats were obtained from the Xian Jiaotong University Animal Center (SCXK (shaan) 2018–003, Xian, China). Rats were housed in the Xizang Minzu University Laboratory Animal Center with a 12 h–12 h light-dark cycle. They were fed regular chow and purified water ad libitum. The animal experiment was conducted following the internationally accepted laboratory animal use and care principles. It is reviewed by the Ethics Committee of Xizang Minzu University (Ethics Approval No. 20200–7). Effective parts of G.s Maxim were extracted as described in section 2.2. We added water to achieve 6.66 g/kg, 3.33 g/kg, and 1.67 g/kg (calculated by raw drug quantity). Rhodiola capsules were used as the positive control.

The rats were randomly divided into six groups (*n* = 8): (1) Control; (2) Hypoxia; (3) 228 mg/kg body weight Rhodiola capsules + Hypoxia; (4) 6.66 g/kg body weight the G.s Maxim’s ethyl acetate extraction + Hypoxia; (5) 3.33 g/kg body weight the G.s Maxim’s ethyl acetate extraction + Hypoxia; and (6) 1.67 g/kg body weight the G.s Maxim’s ethyl acetate extraction + Hypoxia. Rats in control groups were maintained in normal conditions; rats in medication groups were intragastric ally administered compounds for 15 consecutive days.

After the final administration, all rats except those in the control group were placed in a hypobaric oxygen chamber (7000 m, 24 h). At the end of modelling, rats were anesthetized by intraperitoneal injection of urethane; then, brain tissues were removed for pathological examination. We measured serum levels of TNF-α, IL-6, and NF-κB and brain expression of HIF-1α, p65, Bax, and Bcl-2.

The brain tissue was fixed in 10% formaldehyde solution for 12 h, then dehydrated, made transparent, and embedded in paraffin. After sectioning, the specimens were stained with hematoxylin and eosin (HE) and observed under a light microscope. The levels of TNF-α, IL-6, and NF-κB in serum were measured using ELISA according to the manufacturer’s instructions. Western blotting was performed as follows: The total protein of cerebrum samples was extracted in RIPA lysis buffer. According to molecular weight, proteins in brain tissue were separated by SDS-PAGE. Proteins were transferred to polyvinylidene difluoride membranes, blocked with 5% skim milk for 3 h, and incubated with the corresponding primary antibodies overnight at 4 °C. After washing in buffer, the membranes were incubated with a conjugated secondary antibody for 1 h at room temperature. Finally, the membranes were exposed to ECL reagent, and bands were detected using the Image Lab detection system. The intensity of each band was analyzed using Image J software.

### Molecular docking

The crystal structure of core targets (JUN, TNF, TP53, AKT1, HIF-1α, NF-κB) was obtained from RCSB Protein Data Bank (http://www.rcsb.org/), and their corresponding PDB IDs were as follows: 6NOA, 1TNR, 6IUA, 5AAR, 4H6J and 1RAM [20–25]. MOL2 format of active compounds was obtained from the TCMSP database, and their corresponding MOL IDs were as Table 2. Auto Dock Tools Version 1.5.6 (http://mgltools.scripps.edu) and Pymol (https://pymol.org/2/) were applied for molecular docking.

Auto Dock Tools generated and optimized all 3D structures of ligands and proteins. These crystal structures were imported into Auto Dock Tools software for dehydration, hydrogenation, and isolation of original ligands. The optimized targets were constructed in a docking grid box, and the active site of molecular docking was determined using the ligand coordinate in the target protein complex [26]. Finally, molecular docking experiments selected the best affinity conformation as the final docking conformation.

### Statistical analysis

Results were expressed as the mean ± SD. Analysis of variance was performed using GraphPad Prism 8.01. Significant differences between groups were defined as *p* < 0.05. Density analysis of the western blotting bands was performed using Image J software.

## Results

**Fig. 1:**
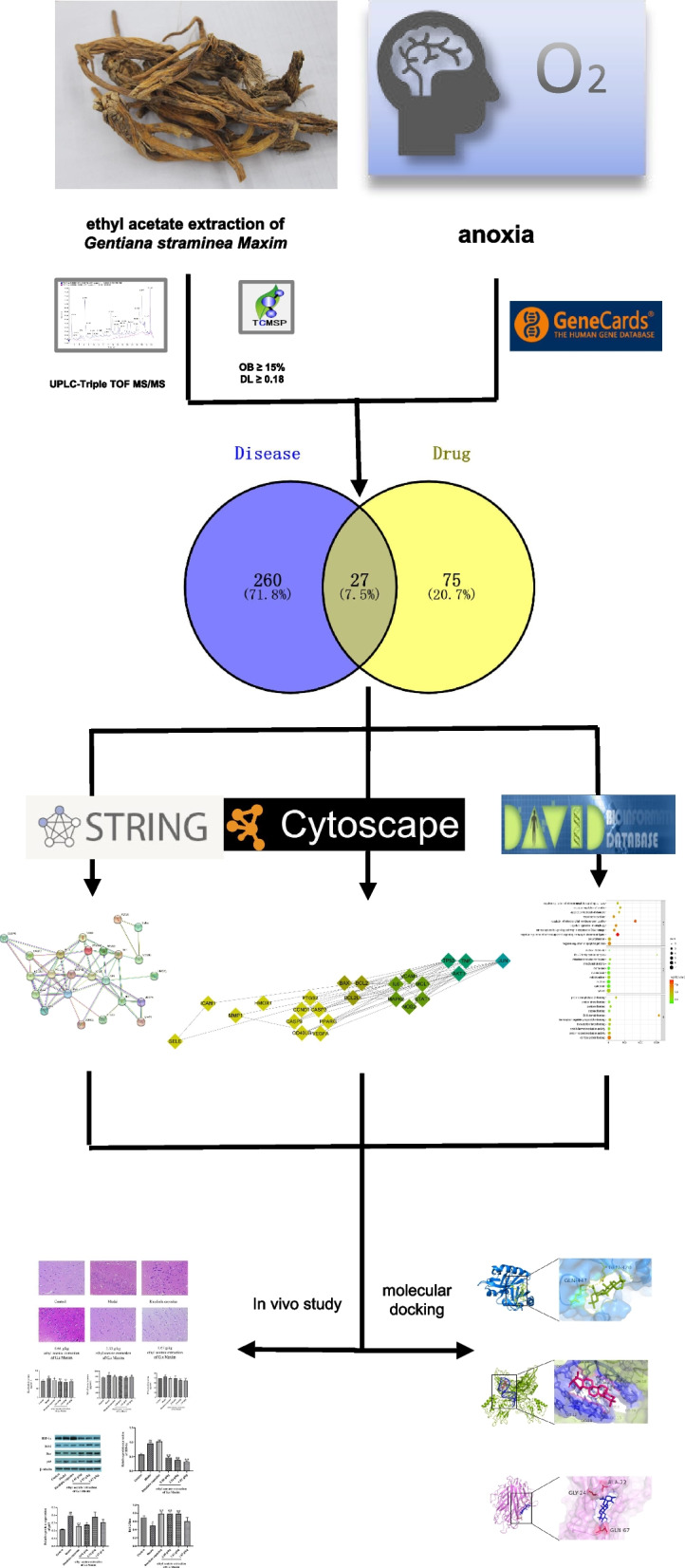
The flowchart of the study

The flowchart of the study is presented in Fig. 1.

### Compound identification

**Fig. 2:**
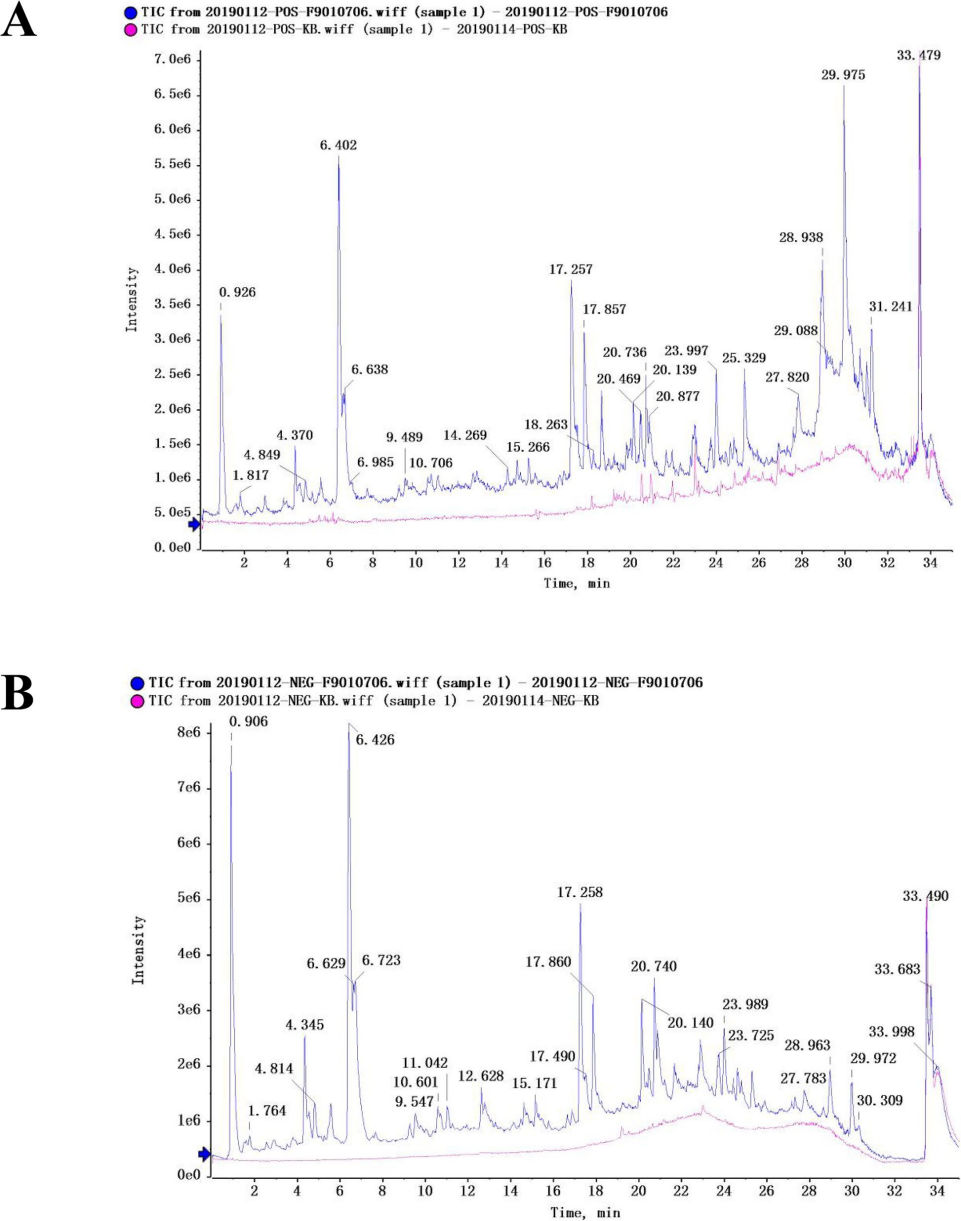
Total ion chromatograms of the G.s Maxim ‘s ethyl acetate extraction in ESI+ and ESI- modes

We identified 20 compounds in the ethyl acetate extraction using UPLC-Triple TOF MS/MS (Fig. 2). Compound names, retention times, found masses, and relative content are displayed in Table 1.

**Table 1:** The chemical constituents and related information of ethyl acetate extract of G.s Maxim

	Name	Formula	Adduct	Found Mass	Error	RT (min)	fragments values
1	gentiobiose	C_12_H_22_O_11_	-H	341.10863	−0.9	0.9	89.0276,119.036,179.0562
2	Swertiamarin [27]	C_16_H_22_O_10_	-H	373.11309	−2.5	4.17	89.03,123.05,149.06,167.07,211.06,211.07
3	Loganin acid [27]	C_16_H_24_O_10_	-H	375.12923	−1.2	4.35	151.0771, 213.0771, 375.1288
4	morroniside	C_17_H_26_O_11_	-H	405.1391	−2.7	4.82	141.0561,155.0350,179.0315,243.0869
5	Rhodopanolic acid	C_10_H_8_O_4_	+H	193.04921	−1.7	5.38	65.0428,91.0561,119.0505,147.0454
6	glucose	C_6_H_12_O_6_	-H	179.05685	4.1	6.34	179.0569
7	6-O-β -D-glucosyl gentiopicrin	C_22_H_30_O_14_	+H	519.17004	−1.5	6.4	129.1549,59.0195,149.0592,189.0553
8	gentiopicroside [27]	C_16_H_20_O_9_	-H	355.10255	−2.5	6.42	59.0194,149.0592,175.0388,189.0553
9	chiratin	C_16_H_22_O_9_	+H	359.13267	−2.8	6.66	111.0816,127.0394,197.079
10	Sweroside [27]	C_16_H_22_O_9_	+H	359.13267	−2.8	6.66	111.0816,127.0394,197.079
11	macrophylloside D	C_25_H_34_O_15_	-H	573.18082	−2.9	12.28	159.0814,203.0710,221.0627,323.0979
12	Saponiflorin	C_27_H_30_O_15_	-H	593.14941	−3	16.1	141.02,153.02,339.0702,409.1114,593.1495
13	luteolin [28]	C_15_H_10_O_6_	+H	287.05461	−1.4	16.52	65.04,171.04,153.02,195.03,269.04,287.06
14	kaempferol [29]	C_15_H_10_O_6_	+H	287.05461	−1.4	16.52	65.04,153.02,195.0281,269.0359,287.0547
15	corosolic acid [30]	C_30_H_34_O_15_	+H	473.3602	−4.9	24.66	121.1013,189.1636,205.1572,203.1782,187.1467,177.1636,409.3463,437.3457
16	Eel rattan acid	C_12_H_12_O_3_	+H	205.08555	−1.8	25.38	65.0417,93.0341,121.0277,149.0226,148.56
17	oleanolic acid	C_30_H_48_0_3_	-H	455.35188	−2.6	27.82	455.3512
18	ursolic acid	C_30_H_48_0_3_	-H	455.35188	−2.6	27.82	455.3512
19	Isovitexanthin [31]	C_27_H_42_0_3_	-H	413.30533	−1.9	29.98	123.0829, 341.2836, 413.3035
20	palmitic acid [32]	C_16_H_32_O_2_	-H	255.23319	0.9	30.09	219.8452

### DLCs from the ethyl acetate extraction

We searched for the G.s Maxim’s ethyl acetate extraction using the TCMSP database and found eight active ingredients had OB ≥ 15% and DL index ≥0.18. These are potential bioactive compounds, including β-sitosterol (MOL005508), ursolic acid (MOL000511), kaempferol (MOL000422), and others (Table 2).

**Table 2:** Characteristics of active ingredients in ethyl acetate extract of G.s Maxim

Number	Mol ID	Compound	CAS	OB (%)	DL
1	MOL005508	corosolic acid	4547-24-4	15.86	0.74
2	MOL000511	ursolic acid	77–52-1	16.77	0.75
3	MOL003166	Swertiamarine	17,388–39-5	21.9	0.42
4	MOL000646	Gentiopicrin	20,831–76-9	22.98	0.39
5	MOL002322	isovitexin	61,838–34-4	31.29	0.72
6	MOL000263	oleanolic acid	508–02-1	29.02	0.76
7	MOL000006	luteolin	491–70-3	36.16	0.25
8	MOL000422	kaempferol	520–18-3	41.88	0.24

### The core anti-hypoxia targets of the ethyl acetate extraction

**Fig. 3:**
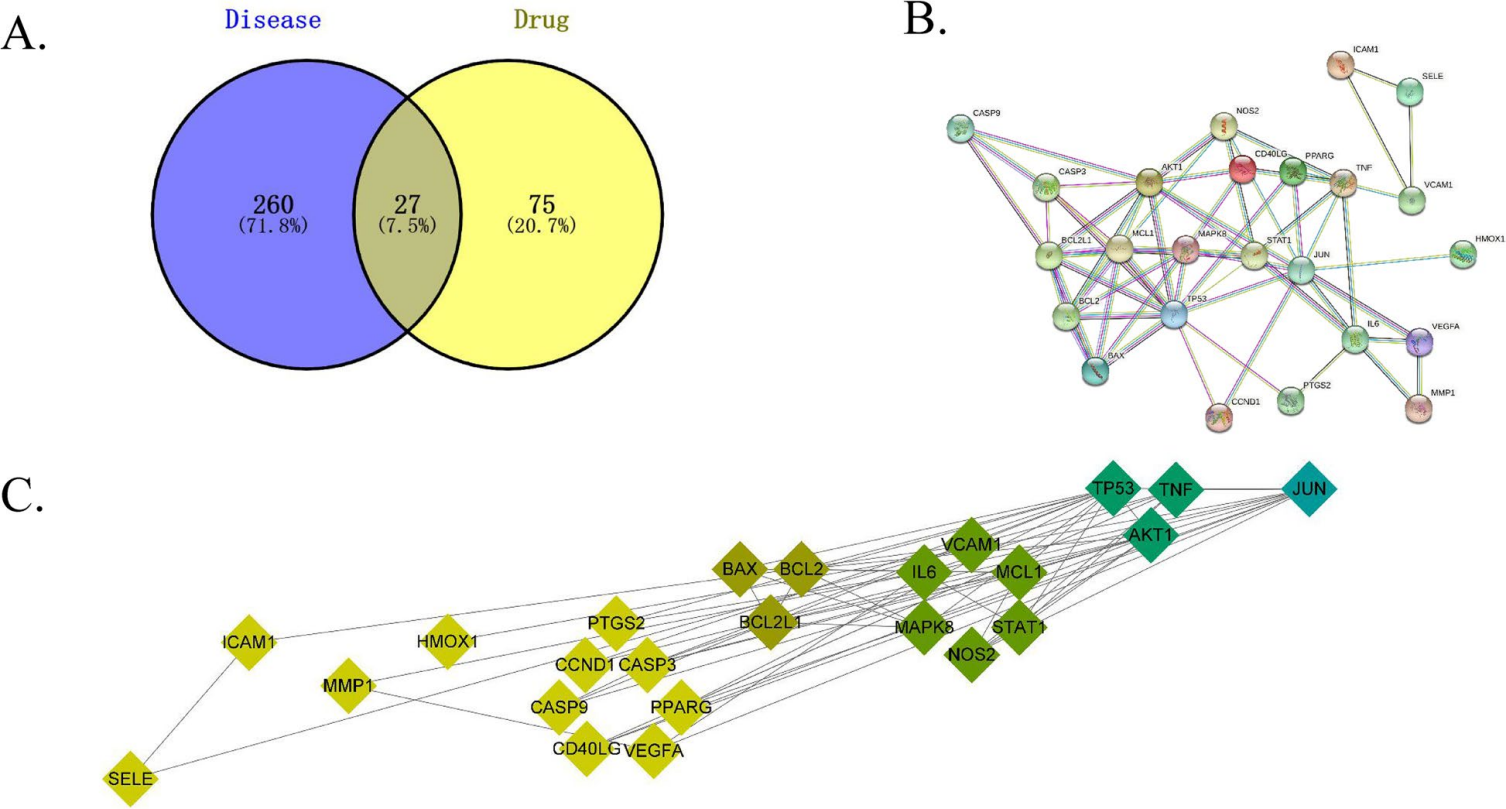
Potential target genes and PPI network map of the G.s Maxim ‘s ethyl acetate extraction therapy for hypoxia. **A** The Venny results of potential target genes of the G.s Maxim ‘s ethyl acetate extraction therapy for hypoxia. **B** The PPI network map of 27 target genes. **C** The core anti-hypoxia targets of the G.s Maxim’s ethyl acetate extraction

The DLC and hypoxia disease targets were analyzed using Wayne analysis, and 27 anti-hypoxia targets were obtained (Fig. 3A). The PPI network for the anti-hypoxia targets is displayed in Fig. 3B (three disconnected targets were removed in the network). Calculation and analysis using Cytoscape 3.8.2 revealed that JUN, TNF, TP53, and AKT1 were essential nodes in the network (Fig. 3C).

### GO and pathway enrichment analysis

**Fig. 4:**
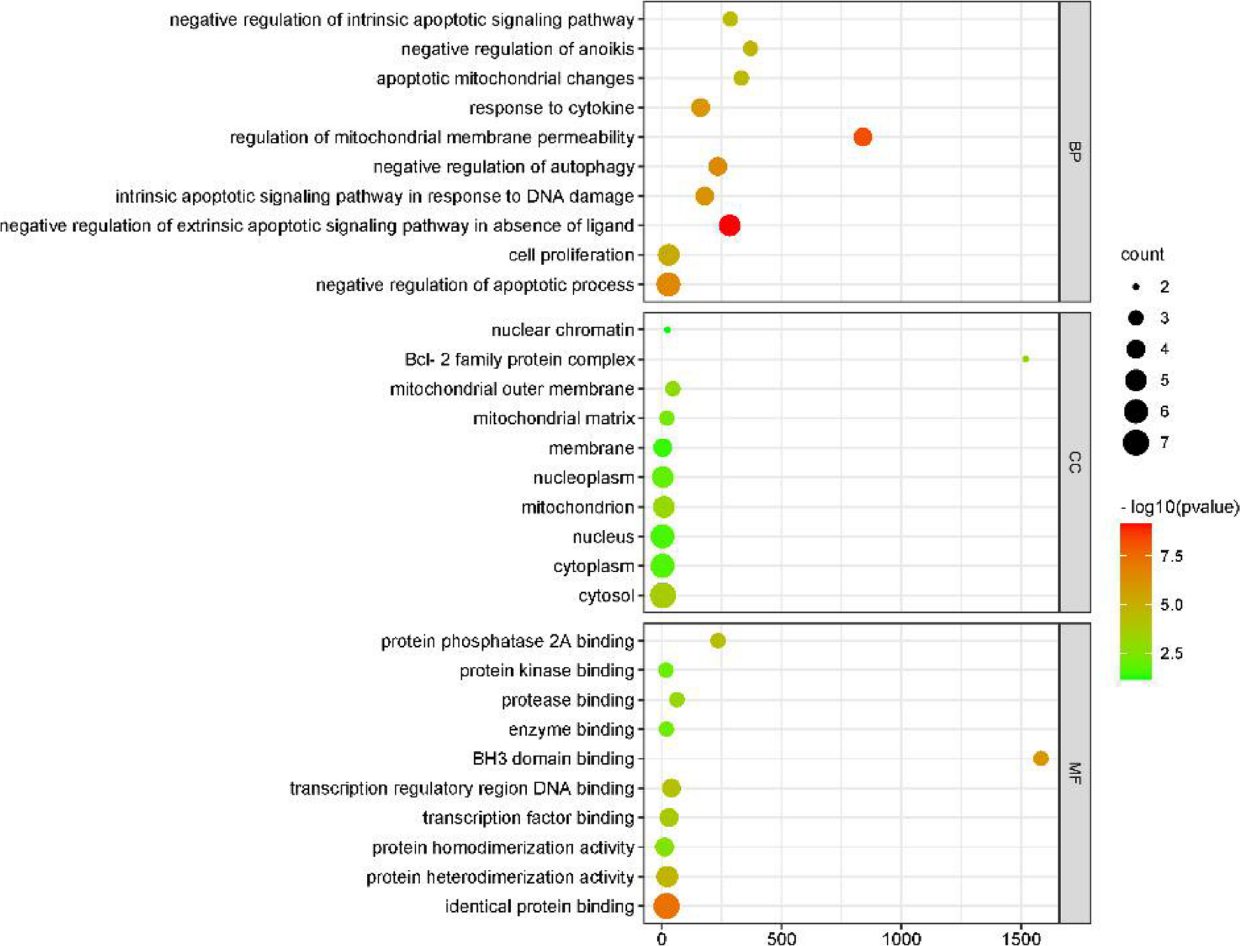
The gene ontology (GO) enrichment analysis for key targets

GO annotation and pathway enrichment analyses were conducted to identify the potential biological functions of targets. The top ten significantly enriched biological process, cell component, and molecular function categories are displayed in Fig. 4. The possible biological processes are related to drug responses, bidirectional regulation of apoptotic processes, positive regulation of neuron apoptotic processes, cellular responses to hypoxia, and cellular responses to cytokine to hypoxia. These genes are involved in cell components, including the Bcl-2 family protein complex, the extracellular space, the cytoplasm, mitochondria, and membranes. The molecular function of these genes correlated with enzyme binding, identical protein binding, protein binding, transcription factor binding, protein homodimerization activity, protein heterodimerization activity, TNF receptor binding, and protein kinase binding.

**Fig. 5:**
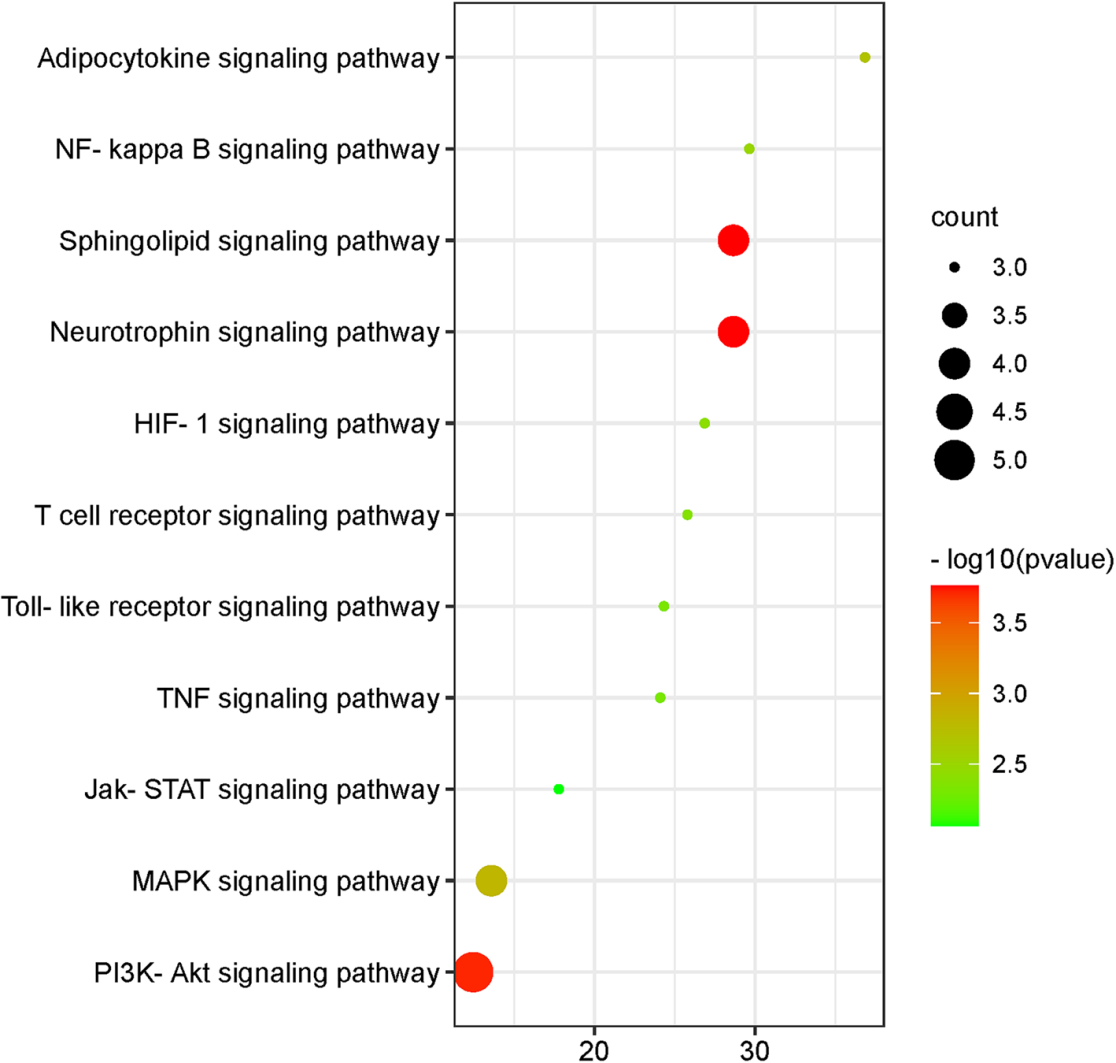
The KEGG pathway enrichment analysis of key targets

KEGG pathway analysis was performed to determine the possible mechanisms of action (Fig. 5). This analysis showed that many target genes were associated with inflammatory-related signaling pathways, including the interleukin-17 signaling pathway, the TNF signaling pathway, the NF-κB signaling pathway, the Toll-like receptor signaling pathway, the T cell receptor signaling pathway, and the AKT signaling pathway. It is regulated by the HIF-1 signaling pathway and the apoptotic signaling pathway associated with HIF-1.

### Protective effect of the G.s Maxim’s ethyl acetate extraction in hypoxic rats

**Fig. 6:**
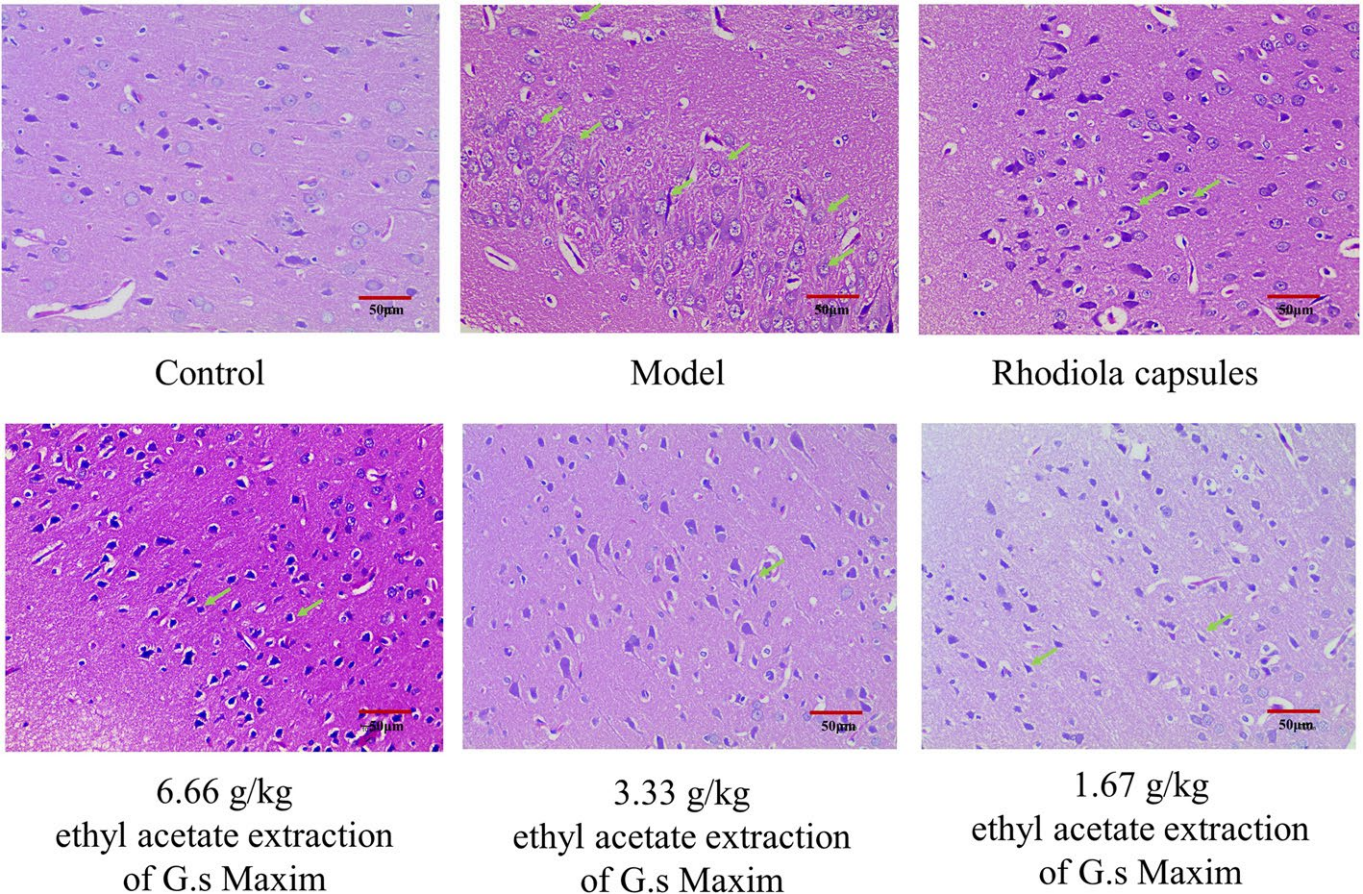
Representative H&E photomicrographs of brain tissue sections from rats exposed to 7000 m low pressure anoxic environment

The effect of each group on brain morphology is presented in Fig. 6. In the hypoxia group, the brain tissues were loose and edematous. The brain cells were disordered, and the cytoplasm was uneven. Neurons in the CA1 region were denatured. There were fewer neurons and a few horizontal axis structures. Compared with the hypoxia group, the brain tissue structure and cell status of G. Maxim and Rhodiola group were normal. Pyramidal cells in the CA1 region of the hippocampus were oval, with sparse nuclear chromatin and evident nucleoli.

**Fig. 7:**
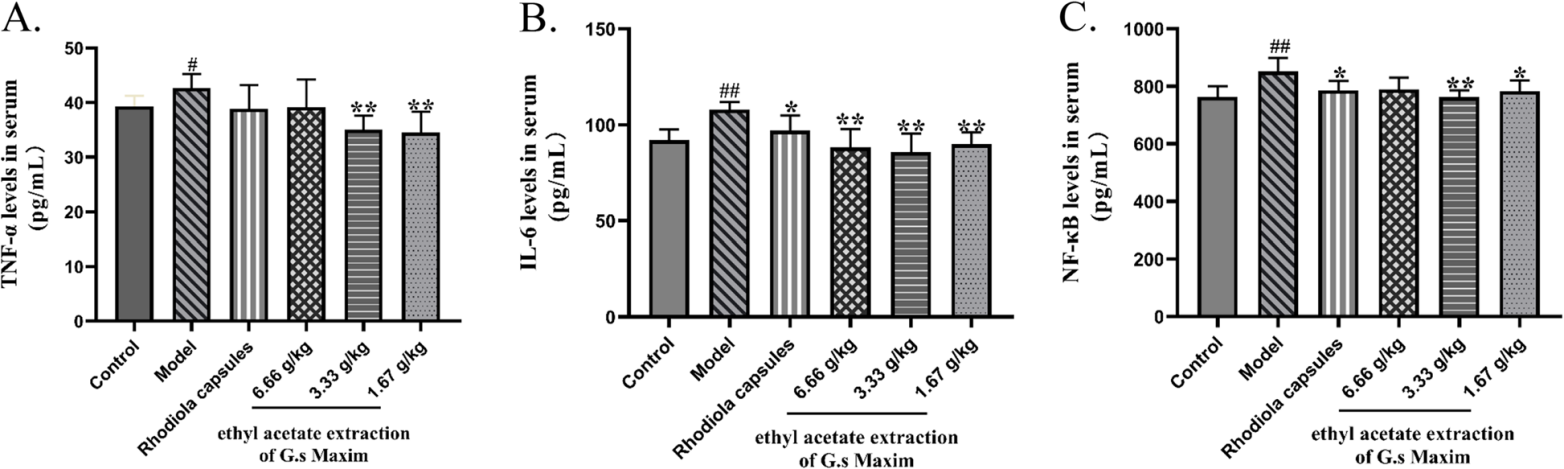
G.s Maxim ethyl acetate extraction administration reduced levels of TNF-α, IL-6, and NF-κ B in serum from rats exposed to 7000 m low pressure anoxic environment. Bars represent the mean ± S.D. from three independent experiments; compare with control group, #*p* < 0.05, ##*p* < 0.01; compare with model group, *p < 0.05, **p < 0.01

Serum TNF-α, IL-6, and NF-κB levels were elevated in the hypoxia group (Fig. 7A-C). G.s Maxim ethyl acetate extraction administration reduced levels of these inflammatory factors. This activity is consistent with positive drug action; the medium-dose group had the most significant effect.

**Fig. 8:**
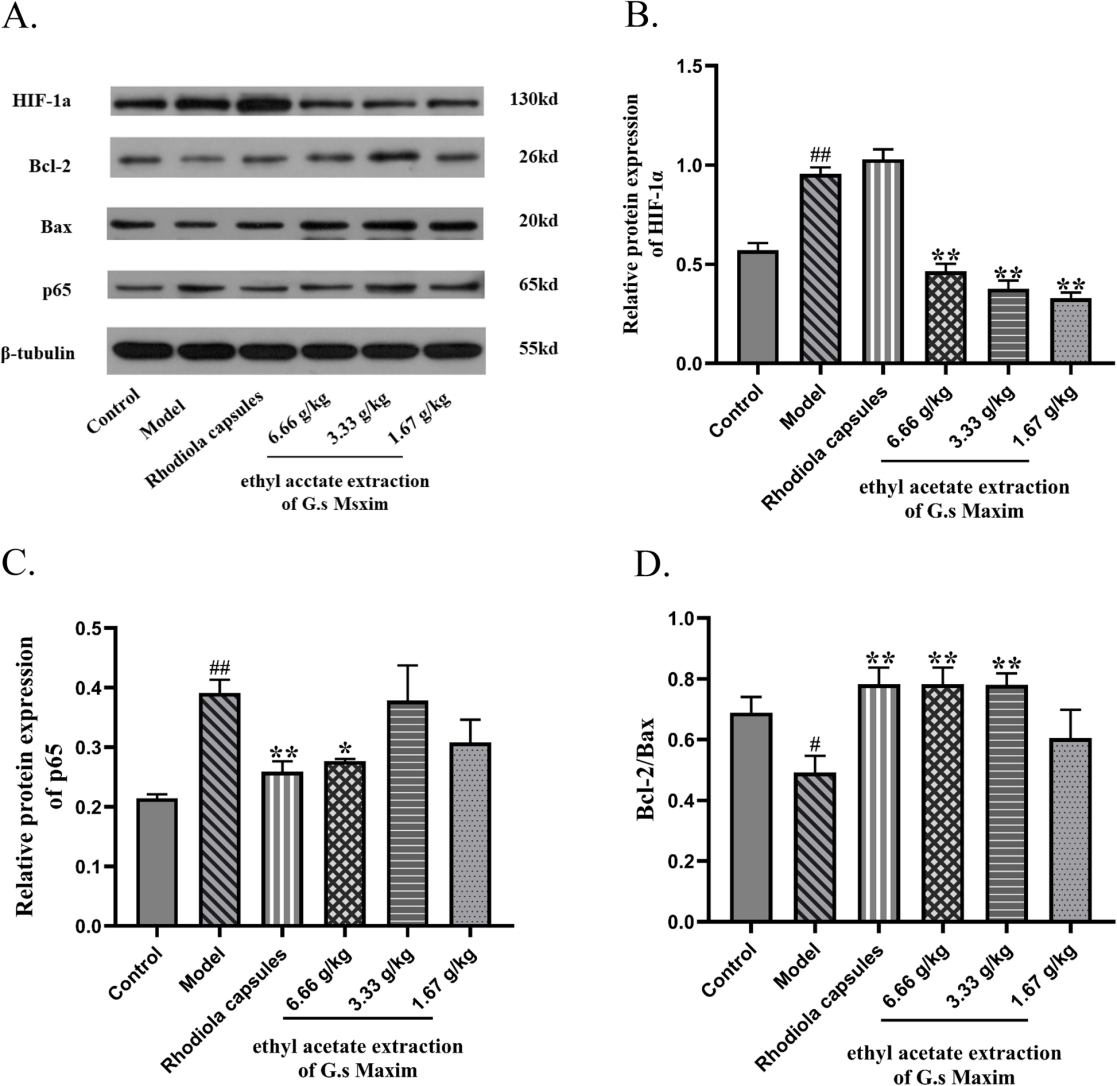
Effect of the G.s Maxim ‘s ethyl acetate extraction on the HIF/NF-KB signaling pathways and hypoxia-induced apoptosis. **A**. The protein levels of HIF-1α, p65, Bcl-2 and Bax were measured by western blotting; **B**. Relative protein level of HIF-1α; **C**. Relative protein level of p65; **D**. The ratio of the relative protein levels of Bcl-2 to Bax. β-tubulin served as a loading control. Bars represent the mean ± S.D. from three independent experiments; compare with control group, #*p* < 0.05, ##*p* < 0.01; compare with model group, *p < 0.05, **p < 0.01

Compared to the control group, brain expression of HIF-1α and p65 was significantly greater in the hypoxic group. Pretreatment with the ethyl acetate extraction downregulated HIF-1α and p65 protein expression. To determine the anti-apoptotic effect, we measured apoptosis-related proteins, including Bcl-2 and Bax. Hypoxia significantly decreased the Bcl-2/Bax ratio in the brains of hypoxic rats. The ethyl acetate extraction of G.s Maxim ethanol extract pretreatment reversed these effects (Fig. 8A-D).

### Molecular docking

**Fig. 9:**
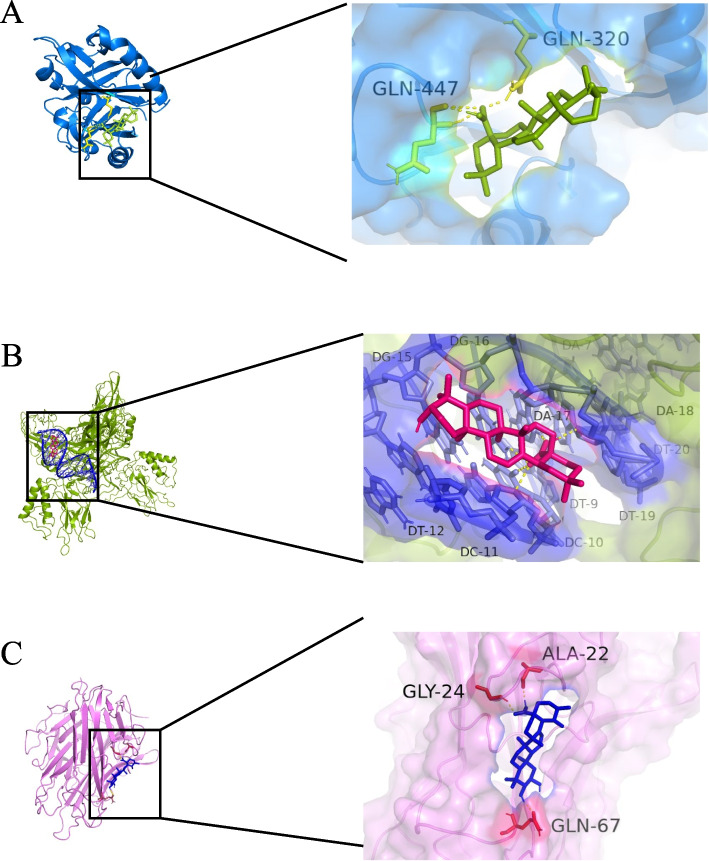
**A** MDT of oleanolic acid on HIF-1α **B** MDT of corosolic acid on NF-κB **C** MDT of oleanolic acid on JUN

In the hypoxia model, the importance of HIF-1α and p65 has been demonstrated, and TNF, TP53, AKT, and JUN were found to be essential nodes in KEGG pathway analysis and the PPI network (scores > 0.9). Molecular docking for eight DLCs and six proteins was analyzed; corosolic, oleanolic, and ursolic acid had a strong affinity with core target proteins (Table 3). Here, the molecular docking of tightly bound compounds and targets is visualized (binding energy of < − 7.0 kcal·mol^− 1^), as shown in Fig. 9A-C. The redocking RMSD values were 0.001, 0.001, and 0.00 Å, respectively.

**Table 3:** Molecular docking results

	JUN	TNF	TP53	AKT1	HIF-1α	NF-κB
luteolin	−6.85	−6.09	−4.91	−5.62	−5.99	−5.66
oleanolic acid	−8.78	−7.39	−6.47	−6.37	−8.23	−6.92
kaempferol	−6.48	−5.31	−4.83	−5.06	−6.51	−5.71
ursolic acid	−8.81	−6.36	− 6.81	− 6.26	−7.89	−6.27
gentiopicroside	−3.62	−4.42	−3.64	−4.32	−6.76	− 4.58
Isovitexanthin	−4.76	− 4.89	−4.22	− 4.4	−4.25	− 3.89
Swertiamarin	− 3.97	− 3.13	− 3.59	−3.27	− 4.25	−2.5
corosolic acid	− 8.32	− 7.55	− 7.05	− 6.99	−8.14	−6.14

The molecular docking results identified regulatory proteins involved in inflammatory responses. These findings and the supporting literature suggest that hypoxic injury is related to inflammatory responses.

## Discussion

High-altitude hypoxia damages the brain, lungs, heart, and other vital organs. Signs and symptoms include headache, acute mountain disease, pulmonary edema, and other manifestations. Currently, nimodipine and sulfadiazine are used as anti-hypoxia medications. Although these medications can relieve the effects of hypoxia, they are associated with toxicities [33]. G.s Maxim is a Tibetan medicine grown on a plateau; it has sounded anti-hypoxia effects and few toxicities. We found 20 constituents of the G.s Maxim’s ethyl acetate extraction using UPLC-Triple TOF MS/MS. The pharmacology network analysis showed that the core targets were JUN, TNF, TP53, and AKT1. These compounds might exert anti-hypoxia effects via the HIF/NF-κ B signaling pathway. We established a hypoxia rat model and used molecular docking to test these findings.

HIF-1 induces cells to adapt to hypoxic environments. Hypoxia blocks the hydroxylation of HIF-1α, resulting in HIF-1 overexpression. After binding to downstream target genes, HIF-1 mediates hypoxia-induced inflammatory responses, apoptosis, oxidative stress, and other functions [34]. Studies demonstrated that NF-κB is a direct regulator of HIF-1α expression. Inhibition of the NF-κB and HIF-1α signaling pathways inhibited the expression of pro-inflammatory cytokines in rats with acute hypoxia-induced brain injury [35–37]. One of the DLCs from the ethyl acetate extract of G.s Maxim (corosolic acid) might exert anti-inflammatory effects by directly inhibiting the expression of TNF-α, IL-6, NF-κB, and other inflammatory factors and by inhibiting NF-κB expression from reducing lipopolysaccharide-induced macrophage inflammation in RAW264.7 mice [38, 39]. Oleanolic and ursolic acids are isomers. Oleanolic acid can be used to treat acute jaundice hepatitis and viral hepatitis. Zhang et al. studied the anti-inflammatory and anti-allergic effects of oleanolic and ursolic acids and found that oleanolic and ursolic acids inhibit types I–IV allergy and various inflammatory animal models [40]. Wang et al. found that swertiamarin may play a protective role in oxygen-glucose deprivation reperfusion injury of PC12 cells by anti-oxidative stress injury and cell apoptosis [41]. These findings suggest that the G.s Maxim’s ethyl acetate extraction may exert anti-inflammatory effects by acting on NF-κB, TNF, and other related targets.

Hypoxia induces the expression of HIF-1 α, caspase-3, Bcl-2, and other proteins in rat models of high-altitude hypoxia; it promotes apoptosis, leading to brain injury [42]. HIF-1 can aggravate apoptosis by blocking p53 transport and modulating the expression of Bcl2/adenovirus E1B interaction protein [43, 44]. Regulating apoptosis may alleviate the damage caused by hypoxia. Cheng et al. found that corosolic acid regulated the expression of anti-apoptotic factors p65 and Bcl-2 and pro-apoptotic factors IκBα and Bax in human gastric cancer cells. Corosolic acid can regulate caspase-3-mediated apoptosis in CT-26 cells [45, 46]. Oleanolic and ursolic acids also regulate apoptosis. Lianqing et al. found that oleanolic acid reduced the expression of caspase3 and p53 proteins to alleviate hepatic ischemia-reperfusion injury. Ursolic acid regulated apoptosis by regulating NF-κB, the Bcl-2-mediated anti-apoptotic pathway, p53, TNF-α, the caspase-3-mediated pro-apoptotic pathway, and the apoptotic substrate poly-ADP ribose [47, 48]. These findings suggest that the G.s Maxim’s ethyl acetate extraction reduces hypoxia-induced apoptosis by acting on NF-κB, p53, Bax, Bcl-2, and other related targets.

Hypoxic damage is associated with oxidative stress. In an anoxic environment, the balance of oxidation and anti-oxidation is broken, leading to oxidative stress. Humans produce substantial amounts of reactive oxygen species that damage biological macromolecules and lead to HIF-1 accumulation, resulting in functional disorders [49]. In a rat model of cerebral ischemia-reperfusion injury, a free radical scavenger (edaravone) alleviated cerebral injury by inhibiting the production of ROS and HIF-1α [50]. Feng et al. reported that corosolic acid protected against oxidative damage of HAECs by increasing antioxidant enzymes such as superoxide dismutase and glutathione peroxidase. Corosolic acid inhibited antioxidant levels in the myocardium to alleviate oxidative stress injury to myocardial cells in mice with myocardial injury [51, 52]. Oleanolic and ursolic acids also have antioxidant stress effects [53, 54]. These findings suggest that the G.s Maxim’s ethyl acetate extraction may alleviate the damage caused by hypoxia by alleviating oxidative stress.

In addition, luteolin, kahenol, and gentiopicrin also have suitable biological activities. Modern pharmacological studies have found that luteolin, kamanol, and other flavonoids have anti-free radical and anti-inflammatory effects. Studies have found that luteolin can significantly reduce coX-2 expression and LPS-induced inflammatory damage by regulating the NF-κB pathway [55–57]. Chen et al. found that gentiopicrin can protect hypoxic-ischemic brain injury rats by exerting antioxidant effects and regulating energy metabolism [58]. Mao et al. suggested that iridoid glycosides significantly inhibit inflammatory cytokines such as TNF-α and IL-6, possibly through the NF-κB pathway and MAPK pathway [59]. In conclusion, corosolic acid, oleanolic acid, ursolic acid, luteolin, kaneferol, and gentiopicrin, all of the natural monomer compounds derived from Gentiana macrophylla, show suitable biological activities in anti-hypoxia injury.

These studies indirectly verified the efficacy of the active components, core targets, and pathways predicted in our study. To test the anti-hypoxia mechanism of G.s Maxim, we established a high-altitude hypoxia rat model. Histological staining of pathological sections showed that ethyl acetate extraction ameliorated the hypoxia-induced damage of hippocampal nerve cells in the CA1 region. Elevated serum expression of TNF-α, IL-6, and NF-κ B in hypoxic rats was reversed by the ethyl acetate extraction, suggesting that the compounds alleviate hypoxia-induced brain damage in rats via anti-inflammatory mechanisms. The ethyl acetate extraction reduced the expression of HIF-1α and p65, increased the Bcl-2/Bax ratio in rat brain tissue, and reduced hypoxia-induced apoptosis.

A genetic algorithm was selected as the docking algorithm for molecular docking. Lower binding energy correlates with the higher stability of the ligand-receptor bond and a greater likelihood of interaction. Binding energies of < − 5.0 kcal·mol^− 1^ are considered good, and binding energy of < − 7.0 kcal·mol^− 1^ is considered strong affinity. The molecular docking results showed that corosolic, oleanolic, and ursolic acids had a robust binding activity with each core target. Therefore, we believe these compounds may be the main active components of the G.s Maxim’s ethyl acetate extraction.

## Conclusion

The ethyl acetate extraction of G.s Maxim exerts anti-hypoxia effects via several targets and pathways. It ameliorates hypoxia-induced damage by reducing inflammation, oxidative stress, and apoptosis. The mechanism might involve the HIF-1/NF-κ B signaling pathway. Whether or not corosolic acid, oleanolic and ursolic acids in the ethyl acetate extraction of G.s Maxim exert anti-hypoxia effects playing a primary role remains unclear.

However, the limitations of network pharmacology and molecular docking should not be ignored. There are several limitations of network pharmacology and molecular docking as follows. 1) Network pharmacology research is based on massive database. The different experimental conditions may lead to false positive results. And the number of small molecule compounds and their targets that have been evaluated is limited, so it still cannot fully reveal their pharmacological effects. 2) Although the binding of small molecules and proteins can be highly mimicked, the conformation of small molecules and proteins will change in vivo due to due to their flexible structure.

We identified the anti-hypoxia properties of G.s Maxim while displaying the power of network data mining. We hope to provide the basis for preventing and treating altitude hypoxia disease and drug development using G.s Maxim. However, further research is needed due to the limitations of network pharmacology and the complexity of medicinal ingredients.

## Supplementary Information

**Additional file 1.**

## Data Availability

A part of datasets generated and/or analysed during the current study are available in the TCMSP database (http://tcmspw.com/),the Identification, anti-hypoxia targets and MOL2 format of bioactive components in the ethyl acetate extraction; Gene Cards database (https://www.genecards.org/), the hypoxia-related targets; STRING database (https://string-db.org/), PPI analysis; DAVID database (https://david.ncifcrf.gov/summary.jsp),GO and pathway enrichment analysis; RCSB database (http://www.rcsb.org/),the target proteins of bioactive components in the ethyl acetate extraction retrieved from the TCMSP database. The others are included in this published article and its supplementary files. The algorithms used to process the data are available from the corresponding author on reasonable request.

## Abbreviations

G.s Maxim: Gentiana straminea Maxim
DLCs: Drug-like compounds
TNF: Tumor necrosis factor
JUN: Jun proto-oncogene
TP53: Tumor protein p53
AKT1: Threonine kinase 1
HIF-1: Hypoxia-inducible factor 1
NF-κB: Nuclear factor kappa-B
IL-6: Interleukin 6
IL-1β: Interleukin-1β
ISVF: Spray voltage
TCMSP: Traditional Chinese medicine systems pharmacology
OB: Oral bioavailability
DL: Drug-likeness
PPI: Protein-protein interaction
GO: Gene Ontology
KEGG: Kyoto Encyclopedia of Genes and Genomes
