# Supplementary material for: Preliminary verification of the anti-hypoxia mechanism of Gentiana straminea maxim based on UPLC-triple TOF MS/MS and network pharmacology
Source: BMC Complement Med Ther. 2022 Nov 25;22:310. doi: 10.1186/s12906-022-03773-0 (PMC9700950; doi:10.1186/s12906-022-03773-0)
Supplement: Supplementary file 1 — Additional file 1. [file 12906_2022_3773_MOESM1_ESM.pdf]

FIG.8(A) Original WB of β-tubulin, p65, Bax, Bcl-2 and HIF-1α in brain tissues of each group. (From left to right are Control group, Model group, Rhodiola capsules group,6.66 g/kg ethyl acctate extraction of G.s Msxim group, 3.33 g/kg ethyl acctate extraction of G.s Msxim group and 1.67 g/kg ethyl acctate extraction of G.s Msxim)

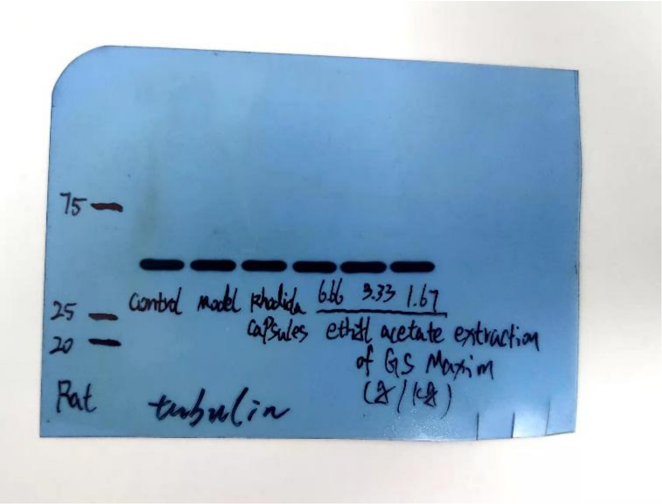

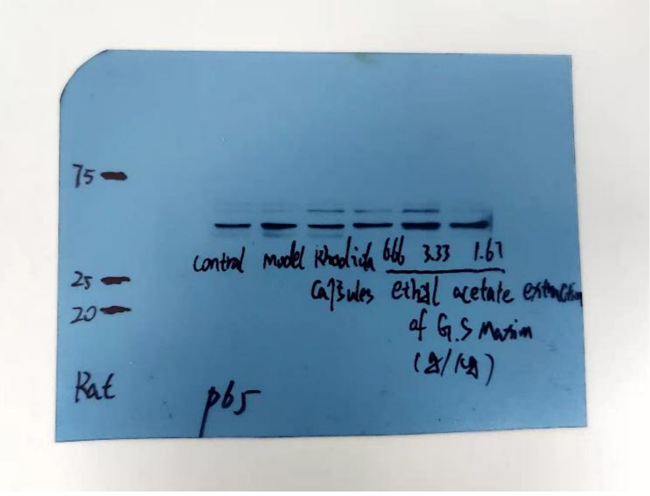

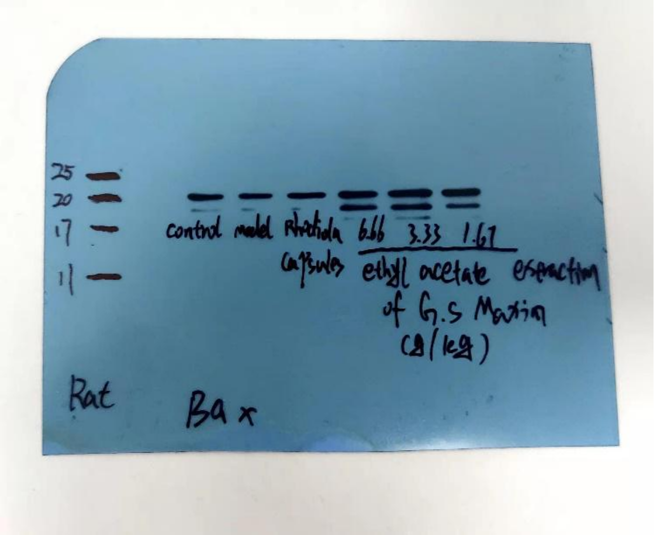
25
20
17
11
control model Rhodiola capsules 6.66 3.33 1.67
ethyl acetate extraction of G.S Maxim (g/kg)
Rat
Bax

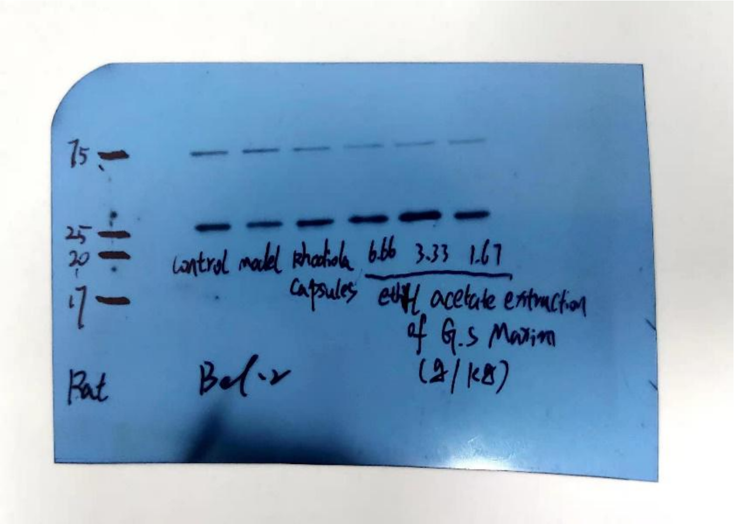
75
25
20
17
control model Rhodiola capsules 6.66 3.33 1.67
ethyl acetate extraction of G.S Maxim (g/kg)
Rat

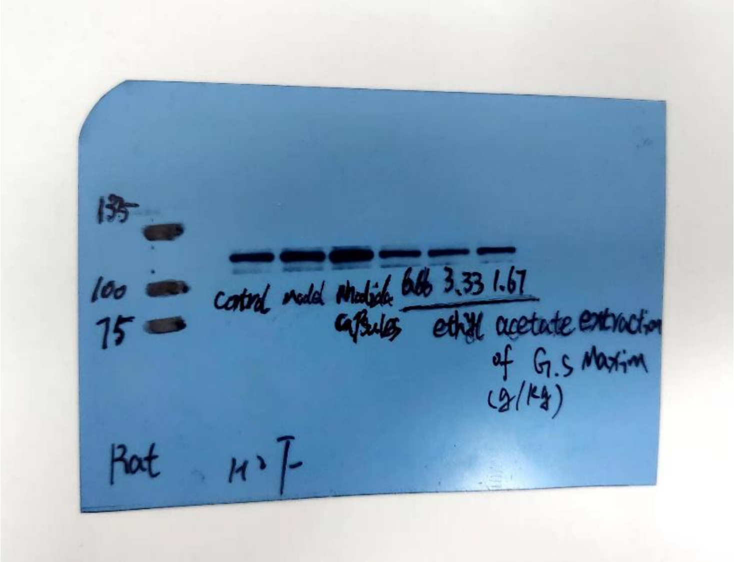
135
100
75
control model Rhodiola capsules 6.66 3.33 1.67
ethyl acetate extraction of G.S Maxim (g/kg)
Rat
